# Mutagenicity and antimutagenicity of six Brazilian *Byrsonima* species assessed by the Ames test

**DOI:** 10.1186/1472-6882-14-182

**Published:** 2014-06-05

**Authors:** Lívia Greghi Espanha, Flávia Aparecida Resende, José de Sousa Lima Neto, Paula Karina Boldrin, Catarine Haidê Nogueira, Mariana Santoro de Camargo, Rone Aparecido De Grandis, Lourdes Campaner dos Santos, Wagner Vilegas, Eliana Aparecida Varanda

**Affiliations:** 1 Department of Biological Sciences, Faculty of Pharmaceutical Sciences of Araraquara, UNESP- São Paulo State University, Rodovia Araraquara-Jaú, km 1, 14801-902 Araraquara, São Paulo, Brazil; 2 Department of Organic Chemistry, Chemistry Institute of Araraquara, UNESP- São Paulo State University, Rua Francisco Degni s/n, Bairro Quitandinha, c.p. 355, 14800-900 Araraquara, São Paulo, Brazil; 3 Campus do Litoral Paulista - Unidade São Vicente, UNESP-São Paulo State University, 11330-900 São Vicente, São Paulo, Brazil

**Keywords:** *Salmonella*/microsome assay, Chemoprevention, Medicinal plants

## Abstract

**Background:**

In various regions of Brazil, several species of the genus *Byrsonima* (Malpighiaceae) are widely used to treat gastrointestinal complications. This genus has about 150 species of shrubs and trees distributed over the entire Neotropical region. Various biological activities have been identified in these plants, especially antioxidant, antimicrobial and topical and systemic anti-inflammatory activities. The aim of this study was to investigate the mutagenicity and antimutagenicity of hydroalcoholic leaf extracts of six species of *Byrsonima: B. verbascifolia, B. correifolia, B. coccolobifolia, B. ligustrifolia, B. fagifolia* and *B. intermedia* by the *Salmonella* microsome assay (Ames test).

**Methods:**

Mutagenic and antimutagenic activity was assessed by the Ames test, with the *Salmonella typhimurium* tester strains TA100, TA98, TA97a and TA102, with (+S9) and without (-S9) metabolization, by the preincubation method.

**Results:**

Only *B. coccolobifolia* and *B. ligustrifolia* showed mutagenic activity. However, the extracts of *B. verbascifolia, B. correifolia, B. fagifolia* and *B. intermedia* were found to be strongly antimutagenic against at least one of the mutagens tested.

**Conclusions:**

These results contribute to valuable data on the safe use of medicinal plants and their potential chemopreventive effects. Considering the excellent antimutagenic activities extracted from *B. verbascifolia*, *B. correifolia*, *B. fagifolia* and *B. intermedia,* these extracts are good candidate sources of chemopreventive agents. However, *B. coccolobifolia* and *B. ligustrifolia* showed mutagenic activity, suggesting caution in their use.

## Background

The use of medicinal plants in folk medicine is based on empirical knowledge gathered for centuries by diverse ethnic groups. This knowledge, based on daily experience and passed down from generation to generation, constitutes the origin of modern medicine [1,2].

*Byrsonima* is a genus of Neotropical trees and shrubs known for yellow, cherry-sized fruits. In various regions of Brazil, several species of this genus are widely used in the treatment of gastrointestinal problems. Research has confirmed a number of different biological activities in these plants, especially antioxidant, antimicrobial and topical and systemic anti-inflammatory activities. Phytochemically, this genus has been noted for the presence of flavonoids and triterpenoids [3].

In previous studies, the methanolic (MeOH) leaf extract of *B. crassa* showed mutagenic activity in the Ames test with *Salmonella typhimurium* strain TA98, without the metabolic activation system (S9), and amentoflavone, one of the metabolites isolated from the ethyl acetate fraction of this species, gave positive results for mutagenicity [4], while the MeOH extract of *B. intermedia* demonstrated signs of mutagenic activity in the *Salmonella* strains TA98 and TA100 [5].

To complement the studies with the genus *Byrsonima*, we also tested MeOH and chloroform extracts of *B. basiloba* for mutagenicity and antimutagenicity. No mutagenic activity was observed in either extract. However, both extracts showed antimutagenic activity against direct and indirect mutagens [6].

Considering the popular use of these plants, the risk of using medicinal extracts without detailed investigation and the valuable role of chemopreventive substances, the aim of this study was to assess the mutagenic activity of hydroalcoholic extracts of leaves of six *Byrsonima* species (*B. verbascifolia*, *B. correifolia*, *B. coccolobifolia*, *B. ligustrifolia*, *B. fagifolia* and *B. intermedia*) by the Ames test, and to identify protective activity in these species against the mutagenicity of the direct and indirect-acting mutagens, 4-nitro- *o*-phenylenediamine (NPD), mitomycin C (MMC), benzo[a]pyrene (B[a]P) and aflatoxin B_1_ (AFB_1_).

## Methods

### Chemicals

Dimethylsulfoxide (DMSO), nicotinamide adenine dinucleotide phosphate sodium salt (NADP), D-glucose-6-phosphate disodium salt, magnesium chloride, L-histidine monohydrate, D-biotin, sodium azide (NaN_3_), 2-anthramine (2-AA), NPD, MMC, 2-aminofluorene (2-AF), B[a]P and AFB_1_ were purchased from Sigma Chemical Co (St. Louis, MO, USA). Oxoid Nutrient Broth No. 2 (Oxoid, England) was used as the bacterial culture medium. D-Glucose, magnesium sulfate, citric acid monohydrate, anhydrous dibasic potassium phosphate, sodium ammonium phosphate, monobasic sodium phosphate, dibasic sodium phosphate and sodium chloride were purchased from Merck (Whitehouse Station, NJ, USA).

### Plant material and extraction

Aerial parts of each species were collected in the states of São Paulo, Tocantins and Piauí, Brazil, as reported in Table 1, which also has details of the voucher specimens.

**Table 1:** Plant material

**Species and identification authority**	**Voucher specimen number**	**Collection site**	**Herbarium (where voucher specimen is deposited)**
*B. coccolobifolia* Kunth.	1397	Itirapina, SP, Brazil	UNICAMP Herbarium
*B. intermedia* A. Juss.	1426	Pratânia, SP, Brazil	UNICAMP Herbarium
*B. correifolia* A. Juss.	27151	José de Freitas, PI, Brazil	Graziella Barroso Herbarium, at Federal University of Piauí
*B. fagifolia* Nied.	743	Porto Nacional, TO, Brazil	Herbarium of the Federal University of Tocantins
*B. verbascifolia* (L.) DC.	481	Porto Nacional, TO, Brazil	Herbarium of the Federal University of Tocantins
*B. ligustrifolia* Mart.	24164	Pratânia, SP, Brazil	Herbarium of São Paulo State University, at Botucatu, SP.

The leaves were separated from the aerial parts, dried at 40°C to constant mass and then pulverized and stored in the dark, under cool, dry conditions, until used. Hydroalcoholic extracts were prepared in 7:3 (v/v) EtOH/H_2_O, in a stainless steel percolator (20 L). The solvent was added to the leaf powder and left standing for 2 h, before packing the percolator, with a solvent:leaf powder ratio of 5:1 (w/w). The percolation was performed at a moderate flow rate of 2 mL/min/kg. The solvent was eliminated from the extract in a rotatory evaporator (Heidolph Laborota 4001), equipped with low- pressure pump control with a Heidolph Rotavac Control valve. The residual water extract was dried in a Micro Module freeze-dryer (Savant Instruments Inc.). The dried extract was powdered and stored in amber bottles at 4°C.

### Mutagenicity test

Mutagenicity was assessed by the Ames test (*Salmonella*/microsome assay), with a preincubation for 20 min, with (+S9) and without (-S9) metabolic activation. The *Salmonella typhimurium* tester strains, TA98, TA100, TA97a and TA102, were kindly provided by Dr. B.N. Ames (Berkeley, CA, USA) [7,8]. These strains were grown overnight from frozen cultures for 12–14 h in Oxoid Nutrient Broth No. 2. The metabolic activation mixture (S9 fraction), prepared from livers of Sprague–Dawley rats treated with the polychlorinated biphenyl mixture Aroclor 1254 (500 mg/kg), was purchased from Molecular Toxicology Inc. (Boone, NC, USA) and freshly prepared before each test. The metabolic activation system consisted of 4% S9 fraction, 1% 0.4 M MgCl_2_, 1% 1.65 M KCl, 0.5% 1 M D-glucose-6-phosphate disodium and 4% 0.1 M NADP, 50% 0.2 M phosphate buffer and 39.5% sterile distilled water [8].

To assay mutagenic activity, five different concentrations of each dry extract (0.20-16.7 mg/plate for *B. verbascifolia*, 0.52-16.7 mg/plate for *B. correifolia,* 0.52-16.0 mg/plate for *B. coccolobifolia*, 0.02-18.0 mg/plate for *B. ligustrifolia*, 0.6-5.0 mg/plate for *B. fagifolia* and *B. intermedia*), dissolved in DMSO, were tested. The extract concentrations were selected on the basis of a preliminary toxicity test. In all subsequent assays, the upper limit of the dose range tested was either the highest non-toxic dose or the lowest toxic dose determined in this preliminary assay. Toxicity was detected either as a reduction in the number of histidine revertants (His+) or as a thinning of the auxotrophic background lawn.

The various amounts of extracts to be tested, dissolved in DMSO, were added to 0.5 mL of 0.2 M phosphate buffer or to 0.5 mL of 4% S9 mixture, plus 0.1 mL of bacterial culture and then incubated at 37°C for 20–30 min. Thereafter, 2 ml of top agar were added, and the mixture poured on to a plate containing minimal agar.

The plates were incubated at 37°C for 48 h and the His + revertant colonies were counted manually. All experiments were done in triplicate. The standard mutagens used as positive controls in experiments without the S9 mix were NPD (10 μg/plate) for TA98 and TA97a, NaN_3_ (2.5 μg/plate) for TA100 and MMC (0.5 μg/plate) for TA102. In experiments with S9 activation, 2-AA (1.5 μg/plate) was used with TA98, TA97a and TA100 and 2-AF (5 μg/plate) with TA102. DMSO (100 μL/plate) served as negative (solvent) control.

### Antimutagenicity test

Only extracts considered non-mutagenic were subject to this test employing the method of preincubation in plates, developed by Maron and Ames [8]. Five different concentrations of extracts (0.013-2.000 mg/plate for *B. verbascifolia*, 0.008-4.000 mg/plate for *B. correifolia,* 0.010-0.500 mg/plate for *B. fagifolia* and 0.007-0.250 mg/plate for *B. intermedia*) were associated with known mutagens in tests with and without metabolic activation, using *S. typhimurium* tester strains TA98, TA100 and TA102. In the tests without metabolic activation, the mutagen NPD (10.0 μg/plate) was used for TA98 and MMC (0.5 μg/plate) for TA102, while in those with metabolic activation, 1.0 μg/plate of B[a]P was used for TA98 and 0.5 μg/plate of AFB_1_ for TA100. The extracts were mixed with 0.5 mL of 0.2 M phosphate buffer, or 0.5 mL of 4% S9 mixture for metabolic activation, 0.1 mL of bacterial culture and the mutagen and incubated at 37°C for 20–30 min. After incubation, 2 mL of top agar was added, and the content of each tube was lightly homogenized and poured onto a plate of glucose minimal agar. After solidification of the top agar, the plates were incubated for 48 h at 37°C, and the number of revertant colonies per plate was counted. The entire assay was performed in triplicate [6,9].

### Data analysis

The mutagenic activity results were analyzed with the statistical software package Salanal 1.0 (U.S. Environmental Protection Agency, Monitoring Systems Laboratory, Las Vegas, NV, from the Research Triangle Institute, RTP, NC, USA), adopting the model of Bernstein et al. [10]. The data (revertants/plate) were assessed by analysis of variance (ANOVA), followed by linear regression. The mutagenicity ratio (MR) was also calculated for each concentration tested; MR is the average number of revertants per test plate divided by the average number of revertants per negative (solvent) control plate. The sample was considered mutagenic when a dose–response relationship was detected and a two-fold increase in the number of revertants (MR ≥ 2) was observed for at least one concentration [7].

The antimutagenic activity results were analyzed with the statistical software GraphPad Prism 5. The data (revertants/plate) were assessed by analysis of variance (one-way ANOVA), followed by Tukey’s test. The antimutagenicity results were expressed as percent inhibition (the ability of the compounds to inhibit the action of the known mutagen), calculated as described by Tachino et al. [11]:

$$\mathrm{Inhibition}\;\left(\%\right)=100‒\left[\left(T/M\right)\times 100\right]$$

where T is the number of revertant colonies in a plate containing mutagen and compounds and M is the number of revertant colonies in a plate containing the mutagen alone.

Results were interpreted as no antimutagenic effect when the inhibition was lower than 25%, a moderate effect for a value between 25% and 40% and strong antimutagenicity for values greater than 40% [9,12].

Cell viability was also determined in each antimutagenesis experiment, to assess the potential bactericidal effect of the mutagens and associations. A sample was considered bactericidal when the number of viable bacterial cells was less than 60% of that observed in the negative control [6,13].

## Results

### Mutagenicity test

Table 2 shows the mean number of revertants/plate (M), the standard deviation (SD) and the mutagenic ratio (MR) after the treatments with the six extracts, observed in *S. typhimurium* strains TA98, TA100, TA97a and TA102, in the presence (+S9) and absence (-S9) of metabolic activation. The results showed that only *B. coccolobifolia* and *B. ligustrifolia* extracts induced an increase in the number of revertant colonies relative to the negative control, indicating mutagenic activity. *B. coccolobifolia*, in the absence of S9, showed activity in the strain TA98, with a dose–response relationship reaching a mutagenic ratio of 2.8. In the presence of S9, in the same strain, at all concentrations tested, this extract reached a mutagenic ratio higher than 2.0. *B. ligustrifolia*, in TA98 without S9, reached a mutagenic ratio of 2.0 at the two highest concentrations tested. None of the other species extracts induced twofold or greater increase in the mean number of revertants relative to the negative control group, in the presence or absence of S9.

**Table 2:** **Mutagenic activity expressed as mean number of revertants/plate ± standard deviation and mutagenicity ratio (in brackets) of hydroalcoholic leaf extracts of six *Byrsonima *species**

**Treatment (mg/plate)**	**TA98**		**Treatment (mg/plate)**	**TA100**		**Treatment (mg/plate)**		**TA97a**	**Treatment (mg/plate)**	**TA102**	
	**- S9**	**+ S9**		**- S9**	**+ S9**		**- S9**	**+ S9**		**- S9**	**+ S9**
***B. verbascifolia***											
**0.0**^**a**^	17 ± 4	68 ± 1	**0.0**^**a**^	113 ± 16	114 ± 11	**0.0**^**a**^	81 ± 6	121 ± 20	**0.0**^**a**^	381 ± 43	341 ± 34
**2.1**	15 ± 3 (0.9)	67 ± 2 (1.0)	**0.4**	91 ± 8 (0.8)	121 ± 12 (1.1)	**0.2**	78 ± 3 (1.0)	137 ± 16 (1.1)	**0.4**	369 ± 3 (1.0)	387 ± 20 (1.1)
**4.2**	24 ± 6 (1.4)	75 ± 5 (1.1)	**0.7**	107 ± 20 (1.0)	124 ± 10 (1.1)	**0.4**	97 ± 2 (1.2)	140 ± 10 (1.2)	**0.7**	305 ± 16 (0.8)	445 ± 32 (1.3)
**8.3**	18 ± 6 (1.0)	81 ± 3 (1.2)	**1.5**	106 ± 2 (0.9)	99 ± 4 (0.9)	**0.8**	117 ± 2 (1.4)	146 ± 7 (1.2)	**1.5**	368 ± 36 (1.0)	353 ± 29 (1.0)
**12.5**	23 ± 3 (1.3)	61 ± 4 (0.9)	**2.2**	105 ± 4 (0.9)	104 ± 5 (0.9)	**1.1**	109 ± 6 (1.3)	166 ± 12 (1.4)	**2.2**	322 ± 45 (0.9)	344 ± 45 (1.0)
**16.7**	29 ± 12 (1.7)	50 ± 1 (0.7)	**3.0**	110 ± 9 (1.0)	93 ± 3 (0.8)	**1.6**	94 ± 5 (1.2)	169 ± 39 (1.4)	**3.0**	373 ± 11 (1.0)	350 ± 27 (1.0)
**C +**	720 ± 28^b^	500 ± 32^c^	**C +**	1375 ± 35^d^	1387 ± 37^c^	**C +**	1115 ± 25^b^	1450 ± 70^c^	**C +**	1468 ± 53^e^	1403 ± 33^f^
***B. correifolia***											
**0.0**^**a**^	20 ± 4	68 ± 1	**0.0**^**a**^	93 ± 6	114 ± 11	**0.0**^**a**^	172 ± 2	99 ± 1	**0.0**^**a**^	352 ± 30	435 ± 43
**2.1**	16 ± 3 (0.8)	86 ± 6 (1.3)	**0.5**	73 ± 3 (0.8)	103 ± 5 (0.9)	**0.5**	185 ± 6 (1.1)	85 ± 4 (0.9)	**0.5**	397 ± 11 (1.1)	608 ± 45 (1.4)
**4.2**	18 ± 2 (0.9)	80 ± 3 (1.2)	**1.0**	90 ± 10 (1.0)	106 ± 8 (0.9)	**1.0**	165 ± 11 (1.0)	123 ± 11 (1.3)	**1.0**	386 ± 33 (1.1)	564 ± 35 (1.3)
**8.3**	27 ± 3 (1.3)	66 ± 6 (1.0)	**2.1**	93 ± 8 (1.0)	96 ± 9 (0.8)	**2.1**	166 ± 18 (1.0)	135 ± 4 (1.4)	**2.1**	413 ± 16 (1.2)	484 ± 33 (1.1)
**12.5**	37 ± 10 (1.8)	62 ± 3 (0.9)	**3.1**	94 ± 11 (1.0)	115 ± 12 (1.0)	**3.1**	186 ± 5 (1.1)	110 ± 4 (1.1)	**3.1**	333 ± 50 (1.0)	354 ± 38 (0.8)
**16.7**	31 ± 6 (1.5)	60 ± 9 (0.9)	**4.2**	65 ± 6 (0.7)	94 ± 4 (0.8)	**4.2**	143 ± 10 (0.8)	134 ± 5 (1.4)	**4.2**	285 ± 21 (0.8)	322 ± 32 (0.7)
**C +**	730 ± 28^b^	500 ± 32^c^	**C +**	1250 ± 35^d^	700 ± 37^c^	**C +**	950 ± 25^b^	1450 ± 70^c^	**C+**	1468 ± 53^e^	1403 ± 33^f^
***B. coccolobifolia***											
**0.0**^**a**^	37 ± 8	24 ± 4	**0.0**^**a**^	112 ± 13	92 ± 9	**0.0**^**a**^	172 ± 2	123 ± 15	**0.0**^**a**^	406 ± 11	341 ± 34
**2.0**	56 ± 3* (1.5)	56 ± 6**(2.3)	**0.5**	115 ± 10 (1.0)	102 ± 12 (1.1)	**0.5**	129 ± 15 (0.7)	144 ± 11 (1.2)	**0.5**	345 ± 8 (0.9)	386 ± 17 (1.1)
**4.0**	61 ± 8* (1.7)	51 ± 1**(2.1)	**1.0**	135 ± 18 (1.2)	115 ± 7 (1.3)	**1.0**	145 ± 10 (0.8)	163 ± 11 (1.3)	**1.0**	338 ± 13 (0.8)	366 ± 12 (1.1)
**8.0**	70 ± 9* (1.9)	67 ± 13*(2.8)	**2.1**	154 ± 25 (1.4)	110 ± 11 (1.2)	**2.1**	175 ± 15 (1.0)	208 ± 29* (1.7)	**2.1**	350 ± 23 (0.9)	330 ± 24 (1.0)
**12.0**	102 ± 10**(2.8)	64 ± 9**(2.6)	**3.1**	170 ± 14 (1.5)	108 ± 8 (1.2)	**3.1**	214 ± 5 (1.2)	215 ± 13**(1.8)	**3.1**	355 ± 13 (0.9)	297 ± 8 (0.9)
**16.0**	67 ± 10* (1.9)	63 ± 13*(2.6)	**4.2**	143 ± 27 (1.3)	115 ± 18 (1.3)	**4.2**	190 ± 7 (1.1)	237 ± 34* (1.9)	**4.2**	358 ± 10 (0.9)	261 ± 2 (0.8)
**C+**	531 ± 28^b^	500 ± 30^c^	**C +**	1375 ± 35^d^	700 ± 38^c^	**C+**	950 ± 25^b^	700 ± 30^c^	**C +**	1143 ± 28^e^	1403 ± 33^f^
***B. ligustrifolia***											
**0.0**^**a**^	18 ± 3	24 ± 4	**0.0**^**a**^	210 ± 33	87 ± 15	**0.0**^**a**^	110 ± 9	115 ± 1	**0.0**^**a**^	262 ± 25	407 ± 30
**2.2**	21 ± 1 (1.2)	22 ± 5 (0.9)	**0.08**	226 ± 13 (1.1)	114 ± 13 (1.3)	**0.16**	106 ± 5 (1.0)	132 ± 12 (1.2)	**0.02**	330 ± 16 (1.3)	379 ± 45 (0.9)
**4.0**	22 ± 1 (1.3)	27 ± 8 (1.1)	**0.16**	211 ± 12 (1.0)	98 ± 6 (1.1)	**0.31**	109 ± 8 (1.0)	136 ± 8 (1.2)	**0.04**	232 ± 2 (0.9)	382 ± 21 (0.9)
**9.0**	20 ± 2 (1.1)	38 ± 7 (1.6)	**0.31**	224 ± 37 (1.1)	90 ± 9 (1.0)	**0.62**	127 ± 7 (1.2)	132 ± 19 (1.2)	**0.08**	244 ± 25 (0.9)	411 ± 20 (1.0)
**13.5**	35 ± 6* (2.0)	34 ± 9 (1.4)	**0.47**	227 ± 24 (1.1)	94 ± 3 (1.1)	**0.94**	90 ± 6 (0.8)	145 ± 17 (1.3)	**0.12**	243 ± 22 (0.9)	395 ± 12 (1.0)
**18.0**	36 ± 5* (2.0)	32 ± 2 (1.3)	**0.62**	209 ± 11 (1.0)	93 ± 16 (1.1)	**1.25**	121 ± 1 (1.1)	133 ± 4 (1.2)	**0.16**	258 ± 11 (1.0)	328 ± 15 (0.8)
**C +**	300 ± 12^b^	227 ± 16^c^	**C +**	919 ± 17^d^	987 ± 32^c^	**C +**	1110 ± 32^b^	1036 ± 41^c^	**C +**	1349 ± 45^e^	1464 ± 43^f^
***B. fagifolia***											
**0.0**^**a**^	23 ± 2	22 ± 3	**0.0**^**a**^	124 ± 10	100 ± 7	**0.0**^**a**^	154 ± 10	164 ± 23	**0.0**^**a**^	321 ± 39	300 ± 58
**0.6**	26 ± 1 (1.1)	28 ± 4 (1.3)	**0.6**	146 ± 13 (1.2)	121 ± 9 (1.2)	**0.6**	190 ± 16 (1.2)	175 ± 21 (1.1)	**0.6**	396 ± 38 (1.2)	269 ± 67 (0.9)
**1.2**	23 ± 3 (1.0)	29 ± 2 (1.3)	**1.2**	127 ± 5 (1.0)	115 ± 3 (1.2)	**1.2**	172 ± 7 (1.1)	176 ± 25 (1.1)	**1.2**	385 ± 31 (1.2)	332 ± 23 (1.1)
**2.5**	27 ± 2 (1.2)	24 ± 8 (1.1)	**2.5**	125 ± 18 (1.0)	126 ± 9 (1.3)	**2.5**	171 ± 16 (1.1)	194 ± 11 (1.2)	**2.5**	356 ± 25 (1.1)	320 ± 54 (1.1)
**3.7**	38 ± 8* (1.7)	25 ± 2 (1.1)	**3.7**	139 ± 23 (1.1)	117 ± 24 (1.2)	**3.7**	176 ± 12 (1.1)	184 ± 6 (1.1)	**3.7**	261 ± 47 (0.8)	273 ± 69 (0.9)
**5.0**	38 ± 6* (1.7)	29 ± 4 (1.3)	**5.0**	155 ± 5 (1.3)	107 ± 10 (1.1)	**5.0**	172 ± 11 (1.1)	176 ± 20 (1.1)	**5.0**	277 ± 64 (0.9)	221 ± 43 (0.7)
**C+**	947 ± 88^b^	767 ± 115^c^	**C+**	1682 ± 98 ^d^	1956 ± 78^c^	**C +**	1766 ± 49^b^	1989 ± 89^c^	**C +**	2656 ± 60^e^	2932 ± 97^f^
***B. intermedia***											
**0.0**^**a**^	22 ± 5	21 ± 8	**0.0**^**a**^	151 ± 14	151 ± 4	**0.0**^**a**^	176 ± 10	249 ± 22	**0.0**^**a**^	303 ± 99	396 ± 12
**0.6**	23 ± 3 (1.0)	21 ± 7 (1.0)	**0.6**	162 ± 7 (1.1)	169 ± 12 (1.1)	**0.6**	187 ± 25 (1.1)	304 ± 28 (1.2)	**0.6**	274 ± 36 (0.9)	427 ± 66 (1.1)
**1.2**	28 ± 4 (1.3)	21 ± 3 (1.0)	**1.2**	165 ± 5 (1.1)	157 ± 15 (1.0)	**1.2**	228 ± 16 (1.3)	315 ± 7 (1.3)	**1.2**	243 ± 19 (0.8)	380 ± 28 (1.0)
**2.5**	30 ± 3 (1.4)	17 ± 6 (0.8)	**2.5**	142 ± 10 (0.9)	160 ± 5 (1.1)	**2.5**	201 ± 26 (1.1)	261 ± 18 (1.0)	**2.5**	285 ± 64 (0.9)	401 ± 60 (1.0)
**3.7**	28 ± 1 (1.3)	25 ± 8 (1.2)	**3.7**	155 ± 18 (1.0)	150 ± 4 (1.0)	**3.7**	199 ± 7 (1.1)	270 ± 19 (1.1)	**3.7**	345 ± 61 (1.1)	351 ± 63 (0.9)
**5.0**	35 ± 5* (1.6)	17 ± 2 (0.8)	**5.0**	163 ± 8 (1.1)	140 ± 7 (0.9)	**5.0**	176 ± 23 (1.0)	261 ± 13 (1.0)	**5.0**	302 ± 53 (1.0)	281 ± 72 (0.7)
**C +**	872 ± 67^b^	736 ± 79^c^	**C +**	1512 ± 64^d^	1500 ± 88^c^	**C+**	1196 ± 52^b^	1217 ± 69^c^	**C+**	2539 ± 187^e^	2114 ± 162^f^

^a^Negative Solvent Control: DMSO, 100 μL/plate; Positive Control (C +): ^b^4-nitro-o-phenylenediamine, 10 μg/plate; ^c^2- anthramine, 1.5 μg/plate; ^d^Sodium azide, 2.5 μg/plate, ^e^Mitomycin C, 0.5 μg/plate; ^f^2-aminofluorene, 5 μg/plate. *p < 0.05; **p < 0.01 (ANOVA).

### Antimutagenicity test

The antimutagenic effect of each extract was assessed from the mean number of revertants/plate, the standard deviation (SD) and the percent inhibition (% I) of the mutagenic activity of NPD, MMC, AFB_1_ and B[a]P on treatment with the five concentrations of the extract. The results are displayed in Table 3. *B. verbascifolia* extract can be considered a strong antimutagen against NPD, as it showed more than 40% inhibition at two of the concentrations tested. When tested with MMC, this extract did not show antimutagenic activity; however, with AFB_1_, all five concentrations tested showed more than 40% inhibition, and concentrations in the range of 0.25-2 mg/plate achieved 91% inhibition, making the extract a very strong antimutagen. Against B[a]P, the five tested concentrations of the extract of *B. verbascifolia* also showed more than 40% inhibition and the highest concentration attained 82%, ranking it as strongly antimutagenic.

**Table 3:** **Antimutagenic activity expressed as mean number of revertants/plate (M) ± standard deviation (SD) and degree of growth inhibition of revertants (%I), in combinations of four *Byrsonima *species hydroalcoholic extracts with direct and indirect mutagens**

**Mutagens (M)**	**NPD**^**b**^		**MMC**^**c**^		**AFB**_**1**_^**d**^		**B[a]P**^**e**^	
**Strains**	**TA 98 (-S9)**		**TA 102 (-S9)**		**TA 100 (+S9)**		**TA 98 (+S9)**	
	**Treatment (mg/plate)**	**M ± SD and (%I)**	**Treatment (mg/plate)**	**M ± SD and (%I)**	**Treatment (mg/plate)**	**M ± SD and (%I)**	**Treatment (mg/plate)**	**M ± SD and (%I)**
*B. verbascifolia*								
	**0**^**a**^	18 ± 4	**0**^**a**^	487 ± 81	**0**^**a**^	114 ± 6	**0**^**a**^	36 ± 5
	**0.013 + M**^**b**^	483 ± 40** (45)	**0.094 + M**^**c**^	1502 ± 138 (6)	**0.125 + M**^**d**^	213 ± 7*** (85)	**0.049 + M**^**e**^	102 ± 24*** (44)
	**0.025 + M**^**b**^	454 ± 60*** (48)	**0.188 + M**^**c**^	1296 ± 24 (19)	**0.250 + M**^**d**^	131 ± 27*** (91)	**0.098 + M**^**e**^	78 ± 6*** (57)
	**0.050 + M**^**b**^	550 ± 41** (37)	**0.375 + M**^**c**^	1366 ± 21 (15)	**0.500 + M**^**d**^	125 ± 16*** (91)	**0.195 + M**^**e**^	85 ± 9*** (54)
	**0.100 + M**^**b**^	628 ± 138* (28)	**0.750 + M**^**c**^	1380 ± 4 (14)	**1.000 + M**^**d**^	132 ± 13*** (91)	**0.390 + M**^**e**^	41 ± 5*** (78)
	**0.200 + M**^**b**^	1001 ± 137	**1.500 + M**^**c**^	1381 ± 58 (14)	**2.000 + M**^**d**^	132 ± 24*** (91)	**0.780 + M**^**e**^	34 ± 7*** (82)
	**M**^**b**^	876 ± 30	**M**^**c**^	1601 ± 226	**M**^**d**^	1399 ± 34	**M**^**e**^	184 ± 8
*B. correifolia*								
	**0**^**a**^	18 ± 4	**0**^**a**^	464 ± 48	**0**^**a**^	104 ± 15	**0**^**a**^	36 ± 5
	**0.013 + M**^**b**^	440 ± 57*** (50)	**0.031 + M**^**c**^	1208 ± 15 (15)	**0.008 + M**^**d**^	353 ± 59*** (60)	**0.250 + M**^**e**^	34 ± 6*** (82)
	**0.025 + M**^**b**^	426 ± 2*** (51)	**0.063 + M**^**c**^	1126 ± 66* (20)	**0.016 + M**^**d**^	248 ± 38*** (72)	**0.500 + M**^**e**^	31 ± 6*** (83)
	**0.050+ M**^**b**^	497 ± 39*** (43)	**0.125 + M**^**c**^	1152 ± 74* (19)	**0.031 + M**^**d**^	370 ± 49*** (58)	**1.000 + M**^**e**^	32 ± 6*** (82)
	**0.100+ M**^**b**^	593 ± 20*** (32)	**0.250 + M**^**c**^	1205 ± 52 (15)	**0.063 + M**^**d**^	180 ± 57*** (80)	**2.000 + M**^**e**^	31 ± 5*** (83)
	**0.200+ M**^**b**^	521 ± 61*** (41)	**0.500 + M**^**c**^	1129 ± 146*(20)	**0.125 + M**^**d**^	206 ± 46*** (77)	**4.000 + M**^**e**^	39 ± 3*** (79)
	**M**^**b**^	876 ± 30	**M**^**c**^	1414 ± 61	**M**^**d**^	883 ± 80	**M**^**e**^	184 ± 8
*B. fagifolia*								
	**0**^**a**^	45 ± 8	**0**^**a**^	355 ± 36	**0**^**a**^	94 ± 12	**0**^**a**^	19 ± 7
	**0.030 + M**^**b**^	705 ± 72 (7)	**0.010 + M**^**c**^	1579 ± 94	**0.030 + M**^**d**^	976 ± 38 (8)	**0.030 + M**^**e**^	378 ± 49*** (41)
	**0.060+ M**^**b**^	612 ± 43 (19)	**0.030 + M**^**c**^	1395 ± 70	**0.060 + M**^**d**^	915 ± 44 (14)	**0.060 + M**^**e**^	405 ± 51*** (37)
	**0.120 + M**^**b**^	623 ± 38 (17)	**0.060 + M**^**c**^	1499 ± 89	**0.120 + M**^**d**^	739 ± 40*** (31)	**0.120 + M**^**e**^	332 ± 35*** (48)
	**0.250 + M**^**b**^	593 ± 51 (21)	**0.120 + M**^**c**^	1432 ± 79	**0.250 + M**^**d**^	781 ± 52** (27)	**0.250 + M**^**e**^	268 ± 43*** (58)
	**0.500 + M**^**b**^	483 ± 40* (36)	**0.250 + M**^**c**^	1440 ± 65	**0.500 + M**^**d**^	680 ± 36*** (36)	**0.500 + M**^**e**^	157 ± 29*** (76)
	**M**^**b**^	755 ± 89	**M**^**c**^	1251 ± 82	**M**^**d**^	1064 ± 101	**M**^**e**^	640 ± 49
*B. intermedia*								
	**0**^**a**^	53 ± 12	**0**^**a**^	317 ± 55	**0**^**a**^	94 ± 12	**0**^**a**^	19 ± 7
	**0.010 + M**^**b**^	677 ± 67* (24)	**0.007 + M**^**c**^	1593 ± 71 (5)	**0.010 + M**^**d**^	981 ± 61 (8)	**0.010 + M**^**e**^	338 ± 37*** (47)
	**0.030 + M**^**b**^	679 ± 61* (24)	**0.010 + M**^**c**^	1584 ± 45 (5)	**0.030 + M**^**d**^	993 ± 32 (7)	**0.030 + M**^**e**^	385 ± 48*** (40)
	**0.060 + M**^**b**^	665 ± 82* (26)	**0.030 + M**^**c**^	1504 ± 68 (10)	**0.060 + M**^**d**^	958 ± 20 (10)	**0.060 + M**^**e**^	293 ± 27*** (54)
	**0.120 + M**^**b**^	632 ± 54** (29)	**0.060 + M**^**c**^	1443 ± 82 (14)	**0.120 + M**^**d**^	864 ± 39** (19)	**0.120 + M**^**e**^	253 ± 19*** (60)
	**0.250 + M**^**b**^	621 ± 42** (30)	**0.120 + M**^**c**^	1409 ± 72 (16)	**0.250 + M**^**d**^	725 ± 23*** (32)	**0.250 + M**^**e**^	148 ± 23*** (77)
	**M**^**b**^	893 ± 79	**M**^**c**^	1672 ± 102	**M**^**d**^	1064 ± 101	**M**^**e**^	640 ± 49

^a^Negative Solvent Control: DMSO, 100 μL/plate; Mutagens (M): ^b^4-nitro-o-phenylenediamine, 10 μg/plate; ^c^Mitomycin C, 0.5 μg/plate; ^d^Aflatoxin B_1_, 0.5 μg/plate; ^e^Benzo[a]pyrene, 1 μg/plate. *p < 0.05, **p < 0.01, ***p < 0.0001 (ANOVA).

*B. correifolia* extract also showed strong antimutagenic activity against NPD, as it induced more than 40% inhibition at four of the five concentrations tested. However, the extract was not antimutagenic against MMC. When mixed with AFB_1_, all five tested concentrations of the extract showed inhibition exceeding 40%, reaching 80% at one concentration. Thus, *B. correifolia* had strong antimutagenic activity against this agent. Associated with B[a]P, all five concentrations of the extract of *B. correifolia* tested showed inhibition of revertants around 80%, reaching 83% at one concentration, representing very high antimutagenicity. Although *B. correifolia* was not found to be antimutagenic against MMC, it demonstrated a potential for significant reduction in the numbers of revertants.

Extract of *B. fagifolia* and *B. intermedia* can be described as moderately antimutagenic against NPD, as they inhibited 36% and 30% of revertants, respectively. Against MMC, these extracts showed no antimutagenic activity. When combined with AFB_1_, these extracts showed 36% and 32% inhibition, respectively, and can be considered moderately antimutagenic. When mixed with B[a]P, the two extracts induced inhibition greater than 40%, reaching 76% and 77%, respectively, and ranking them as strongly antimutagenic.

## Discussion

In Brazil, plants of the genus *Byrsonima* (Malpighiaceae) represent a rich source of catechin and epicatechin derivatives and are used in folk medicine for the treatment of gastric ulcers, inflammation, skin infections, fever and asthma. They are popularly known as “murici-vermelho” or “murici-cascudo” and grow wild in the cerrado (savannah-like) vegetation of Brazil. *Byrsonima* species have been scientifically proven to possess several pharmacological properties, such as antiulcerogenic, mutagenic and antimicrobial activity [14].

The results of this study demonstrate an absence of any mutagenic activity in leaf extracts of *B. verbascifolia*, *B. correifolia*, *B. fagifolia* and *B. intermedia,* at all the concentrations tested on the four *S. typhimurium* strains, since the number of revertant colonies observed on each test plate was less than twice that in the negative control [7]. However, *B. coccolobifolia* extract doubled the number of revertant colonies in strain TA98, both in the presence and in the absence of metabolic activation, suggesting an ability to cause frameshift mutations, before and even after being metabolized. In strain TA97a, the dose–response for *B. coccolobifolia* went up to a mutagenicity ratio of 1.9, giving evidence of mutagenicity. *B. ligustrifolia* also doubled the number of revertant colonies in strain TA98 in the absence of metabolic activation and can be considered an inducer of frameshift mutations.

In a previous study, we showed that a MeOH extract of *B. crassa* exhibited mutagenic activity in the Ames test. The following compounds were isolated from the acetate fraction: quercetin-3-*O*-β-_D_-galactopyranoside, quercetin-3-*O*-α-_L_-arabinopyranoside, amentoflavone, methyl gallate and (+)-catechin. Among these, only amentoflavone exhibited positive mutagenicity. Therefore, this compound contributes to the mutagenic activity observed in the MeOH extract [4].

In another study, MeOH, hydromethanol and chloroform extracts of *B. intermedia* were assessed for mutagenicity by the Ames test and mutagenic activity was not positively identified, in any extract, but the MeOH extract showed signs of mutagenicity to the strains TA98 (+S9,-S9) and TA100 (-S9). The values of the MR were close to 2, and the dose–response effect was significant. Phytochemical analysis of the MeOH extract furnished (+)-catechin, (-)-epicatechin, quercetin-3-*O*-β-_D_-galactopyranoside, methyl gallate, gallic acid, quercetin-3-*O*-α-_L_-arabinopyranoside, amentoflavone, quercetin, quercetin-3-*O*-(2”-*O*-galloyl)- β -galactopyranoside and quercetin-3-*O*-(2”-*O*-galloyl)- α -arabinopyranoside [5].

Comparing the compounds in the MeOH extracts of *B. intermedia* with those from *B. crassa *[15], similar profiles were observed, but a difference was seen in the flavonol concentration and the difference in the amentoflavone content explained the results obtained. In *B. crassa*, mutagenic activity was observed and the main compound of the extract responsible for this effect was amentoflavone [4]. In *B. intermedia,* this biflavonol is also present, but in smaller amounts, explaining the signs of mutagenic activity obtained in the assays with *Salmonella *[5].

Quercetin is a compound that is always present in *Byrsonima* extracts [4-6]. It is a flavonoid known for its mutagenic potential. Resende et al. [16] showed that quercetin is highly mutagenic in TA98, TA100 and TA102. This compound has been observed to induce a mutation ratio of 20.4, suggesting that quercetin contributes to the mutagenic potential of this genus.

For the hydroalcoholic extract of *B. intermedia* and *B. fagifolia,* the results obtained here are also similar to results described for the MeOH extract [5,17]. This fact suggests that the MeOH and hydroalcoholic extracts are similar in composition.

The detection of genotoxicity is highly advisable, so as to avoid the risk of genotoxic exposure to mutagens and carcinogens. However, some genotoxic compounds cannot be completely avoided because they are air pollutants, or some might be ingested as food contaminants. Also, some therapeutic drugs belong to an important group of genotoxic compounds. Antimutagenicity studies have been developed to diminish the risk in the event of genotoxic exposure [18]. There have been several reports in the literature, that medicinal plants or fruit juices have components such as polyphenols, vitamins, chlorophylls, terpenes and unknown organic compounds, which are described as antimutagens and perhaps anticarcinogens [19].

In the present study, the *B. verbascifolia* and *B. correifolia* extracts acted as strong antimutagens against NPD, with inhibition up to 48% and 51% of induced revertants, respectively. *B. fagifolia and B. intermedia* were moderately antimutagenic, and their percentages of inhibition reached 36% and 30%. These extracts were able to prevent frameshift mutations.

When combined with MMC, none of the extracts could be considered antimutagenic, although *B. correifolia* extract reduced significantly the number of revertants at several doses.

Against AFB_1_, *B. verbascifolia* and *B. correifolia* extracts performed as strong antimutagens, reaching inhibition levels of 91% and 80% of revertants, respectively. It should be emphasized that when the concentration of the *B. verbascifolia* extract was varied, the inhibition remained at 91%, which reinforces its strongly protective potential. *B. fagifolia* and *B. intermedia* were moderately antimutagenic in these tests, reaching 36% and 32% inhibition, respectively. This demonstrates the potential of these extracts to be used as protective agents against indirect mutagens, which require metabolic activation.

Against B[a]P, all the extracts showed strong antimutagenicity and may be able to prevent frameshift mutations. *B. verbascifolia* reached a level of inhibition of 82%, *B. correifolia* 83%, *B. fagifolia* 76% and *B. intermedia* 77%. It should be emphasized that even when the concentration of the *B. correifolia* extract was varied, the percentages of inhibition remained very close to each other (at 82-83%), confirming its protective potential.

In previous studies, no mutagenic activity was observed in MeOH and chloroform extracts of *B. basiloba,* however, both extracts showed antimutagenic activity. The highest inhibition level (89%) was obtained with the MeOH extract, in the strain TA100 in the presence of AFB_1._ Phytochemical analysis of these extracts revealed the presence of *n*-alkanes, lupeol, ursolic and oleanolic acid, (+)-catechin, quercetin-3-*O*-α-_L_-arabinopyranoside, gallic acid, methyl gallate, amentoflavone, quercetin, quercetin-3-O-(2”-*O*-galloyl)-β-_D_-galactopyranoside, and quercetin-3-*O*-(2”-*O*-galloyl)-α-_L_-arabinopyranoside [6].

Rinaldo et al. [14] demonstrated that in MeOH extracts and aqueous infusions from the leaves of five *Byrsonima* species, only in *B. coccolobifolia* was it not possible to observe the presence of catechins and epicatechins. In the other four species analyzed, it was found that the MeOH extracts showed larger amounts of catechins than the infusions, per gram of leaves. *B. basiloba* showed the highest concentration of catechin diastereomers, followed by *B. verbascifolia*, *B. crassa* and *B. intermedia*.

The results of this study are in agreement with those in the above mentioned research. *B. verbascifolia* and *B. intermedia* did not exhibit mutagenic activity, nor did *B. basiloba *[6], but all of them were antimutagenic and showed high concentrations of catechins [14], suggesting that catechins are important in antimutagenic activity. This point is corroborated by a study that claims that catechins are already known for their antimutagenic and cancer preventive properties [20,21].

The phytochemistry of all species analyzed to date in the genus *Byrsonima* is similar; we suggest that all species possess amentoflavone and quercetin, but in varied amounts. Probably, *B. coccolobifolia* and *B. ligustrifolia* possess higher concentrations of amentoflavone or quercetin, because they were mutagenic, but all the other species studied here probably possess a lower concentration of these flavonoids.

Although the mutagenic biflavonoid amentoflavone was present in the non-mutagenic *B. basiloba,* the amount of this compound found in the MeOH extract (1.79 mg/g of MeOH extract) was much smaller than that found in the (mutagenic) *B. crassa* (17.04 mg/g of MeOH extract) and *B. intermedia* (13.70 mg/g of MeOH extract) extracts, which showed weak signs of mutagenicity in an earlier study [5,6].

Finally, we have to emphasize the excellent chemopreventive ability of these extracts, especially with respect to compounds that require metabolic activation. All extracts evaluated were considered strongly antimutagenic against at least one of the tested mutagens.

The careful study of medicinal plants should be encouraged, because while many beneficial properties are confirmed or discovered, as shown here, some species may pose risks to users.

## Conclusion

These results contribute valuable data on the safe use of medicinal plants and some benefits, such as chemopreventive effects. Some medicinal plants should be used with caution by the population, such as *B. coccolobifolia* and *B. ligustrifolia*, because they are mutagenic. However, *B. verbascifolia*, *B. correifolia*, *B. fagifolia* and *B. intermedia* were found to be strongly antimutagenic against at least one of the mutagens tested and, given the outstanding antimutagenic activities revealed in some tests, these extracts are good candidates for development as chemopreventive agents. Considering that medicinal herbs contain complex mixtures of thousands of components that can act alone or synergistically [22], it is important to continue phytochemical studies of hydroalcoholic extracts, to provide the chemical profile of the active species.

## Abbreviations

NPD: 4- nitro-o-phenylenediamine; NaN_3_: Sodium azide; MMC: Mitomycin C; 2-AA: 2-anthramine; 2-AF: 2-aminofluorene; DMSO: Dimethylsulfoxide; NADP: Nicotinamide adenine dinucleotide phosphate sodium salt; B[a]P: Benzo[a]pyrene; AFB_1_: Aflatoxin B_1_; +S9: With metabolization; -S9: Without metabolization; MR: Mutagenicity ratio; SD: Standard deviation.

## Competing interests

The authors declare that they have no competing interests.

## Authors’ contributions

LGE designed and performed the experiments, interpreted the results and drafted the manuscript. FAR performed some experiments and interpreted some results. JSLN, WV and LCS prepared the hydroalcoholic extract of *Byrsonima* species. FAR, PKB and CHN participated in the assays of mutagenicity. FAR, MSC and RADG participated in the assays of antimutagenicity. FAR and EAV read the manuscript critically and participated in its revision. All authors have read and approved the final manuscript.

## Pre-publication history

The pre-publication history for this paper can be accessed here:

http://www.biomedcentral.com/1472-6882/14/182/prepub
